# Commentary: p31-43 Gliadin Peptide Forms Oligomers and Induces NLRP3 Inflammasome/Caspase 1- Dependent Mucosal Damage in Small Intestine

**DOI:** 10.3389/fimmu.2019.02792

**Published:** 2019-11-29

**Authors:** Exequiel Barrera, Fernando Chirdo, Sergio Pantano

**Affiliations:** ^1^Biomolecular Simulations Group, Institut Pasteur de Montevideo, Montevideo, Uruguay; ^2^Instituto de Estudios Inmunológicos y Fisiopatológicos (UNLP-CONICET), Universidad Nacional de La Plata, La Plata, Argentina

**Keywords:** coarse grained (CG), simulations, celiac disease, p31-43, gliadin peptides, sirah force field

In our recent publication p31-43 Gliadin Peptide Forms Oligomers and Induces NLRP3 Inflammasome/Caspase 1- Dependent Mucosal Damage in Small Intestine” (1) we showed by a combination of experimental and simulation techniques that the peptide p31-43 Gliadin has an intrinsic propensity to form oligomers, which trigger the NLRP3 inflammasome, resulting in intestinal inflammation and pathology. In particular, molecular simulations performed with the SIRAH force field (2), showed that isolated p31-43 peptides exhibit a broad conformational dynamic with some PPII component, mostly related to the presence of Pro36 and Pro42. Simulation of multiple replicas showed a spontaneous tendency to aggregation with a concomitant increase in the PPII content for Pro38 and Pro 39.

After our paper came out, an independent group published the NMR structure of p31-43 and its P36A and F37A mutants (3). This work presented apparently contrasting results based on NMR spectroscopy suggesting p31-43 Gliadin is mainly monomeric, but not discarding the presence of possible aggregated structures. As the peptide structures were deposited in the Protein Data Bank, we reanalyzed our molecular dynamics trajectory conducting a structural comparison between the conformations sampled in our simulations and those based on NMR. As it can be observed from Figure 1A, the matching observed between monomer simulations and experimental structures is outstanding. Moreover, the structural superposition between individual peptides within the aggregate and the experimental structures is also remarkable (Figure 1B). This provides support to the idea that “the monomers are in fast exchange with self-assembled structures” and that “the 3D models represent the secondary elements adopted also in the oligomeric forms” (3). Indeed, selected conformations from the simulations are indistinguishable from those coming from the experimental determination (Figure 1C).

**Figure 1:**
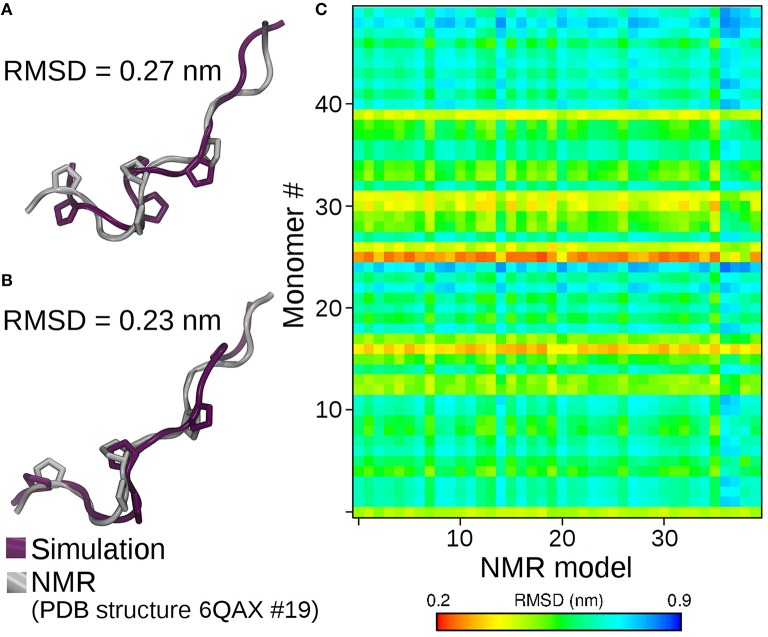
Structural superposition of the best matching solution between NMR derived conformations and simulation of isolated peptides **(A)**, and peptides *within* the 50-mer aggregate **(B)**. On **(C)**, the final structures obtained by molecular dynamics of each peptide forming the oligomer (Y-axis) are compared against all the conformers reported in the NMR family of structures (X-axis). The degree of structural similarity is reported as the root mean square deviation (RMSD) between the C-alpha carbons of simulated and NMR derived conformers. Small RMSD values (red-yellow) identify high structural similarity. Simulations of P36A and F37A showed the same degree of structural similarity (not shown).

Besides highlighting the capacity of our simulation method to characterize the conformational behavior of peptides (Figure 1), the remarkable agreement between simulations and experiments in the oligomeric case provides strong support to the hypothesis that the p31-43 Gliadin peptide suffers very minor conformational changes when passing from monomeric to oligomeric states. This further strengthens the idea that aggregates might work as reservoirs that protect p31-43 from degradation. Though p31-43 monomers are quite resistant to degradation, the self-assembly process extends the persistence of large aggregates with higher toxic potency, particularly when disruption of the normal physiology of internal vesicles (1) and release of danger signals are considered as driving events for inflammation and cell damage (4).

## Author Contributions

EB, FC, and SP wrote the paper.

### Conflict of Interest

The authors declare that the research was conducted in the absence of any commercial or financial relationships that could be construed as a potential conflict of interest.
